# Characterization of ROS Metabolic Equilibrium Reclassifies Pan-Cancer Samples and Guides Pathway Targeting Therapy

**DOI:** 10.3389/fonc.2020.581197

**Published:** 2020-10-20

**Authors:** Shuai Shen, Zihao Yan, Jianqi Wu, Xing Liu, Gefei Guan, Cunyi Zou, Qing Guo, Chen Zhu, Tianqi Liu, Chen Chen, Ling Chen, Peng Cheng, Wen Cheng, Anhua Wu

**Affiliations:** ^1^Department of Neurosurgery, The First Hospital of China Medical University, Shenyang, China; ^2^Key Laboratory of Cell Biology, Ministry of Public Health, Key Laboratory of Medical Cell Biology, Ministry of Education, The Research Center for Medical Genomics, College of Life Sciences, China Medical University, Shenyang, China; ^3^Department of Neurosurgery, Chinese People's Liberation Army of China (PLA) General Hospital, Medical School of Chinese PLA, Institute of Neurosurgery of Chinese PLA, Beijing, China

**Keywords:** pan-cancer, ROS metabolism, multi-omics landscape, ROS-targeted therapy, predictive model

## Abstract

**Background:** Abnormal redox equilibrium is a major contributor to tumor malignancy and treatment resistance. Understanding reactive oxygen species (ROS) metabolism is a key to clarify the tumor redox status. However, we have limited methods to evaluate ROS in tumor tissues and little knowledge on ROS metabolism across human cancers.

**Methods:** The Cancer Genome Atlas multi-omics data across 22 cancer types and the Genomics of Drug Sensitivity in Cancer data were analyzed in this study. Cell viability testing and xenograft model were used to validate the role of ROS modulation in regulating treatment efficacy.

**Results:** ROS indexes reflecting ROS metabolic balance in five dimensions were developed and verified. Based on the ROS indexes, we conducted ROS metabolic landscape across 22 cancer types and found that ROS metabolism played various roles in different cancer types. Tumor samples were classified into eight ROS clusters with distinct clinical and multi-omics features, which was independent of their histological origin. We established a ROS-based drug efficacy evaluation network and experimentally validated the predicted effects, suggesting that modulating ROS metabolism improves treatment sensitivity and expands drug application scopes.

**Conclusion:** Our study proposes a new method in evaluating ROS status and offers comprehensive understanding on ROS metabolic equilibrium in human cancers, which provide practical implications for clinical management.

## Introduction

On a global level, cancer is a dominant and burdensome challenge for modern molecular medicine (1). While many resources have been directed to this area, we still have far to go before we fully understand the cellular and the molecular mechanisms behind tumorigenesis and malignancy progression. In recent years, metabolic abnormalities have been flagged as hallmark phenotypes in most human cancers (2, 3); therefore, considerable efforts have been made to target metabolic vulnerabilities, which hold great promises for cancer treatments (4).

Oxygen metabolism lies at the heart of biology, as it supports energy production, which inevitably generates unstable oxygen derivatives, *i*. textite., reactive oxygen species (ROS) (5). These species are generated either in the mitochondria, via electron escape from the respiratory chain, or in the cytoplasm, via oxygen oxidized by the NOXs families or the DUOXs families. Proper relatively low ROS level is essential for maintaining cellular processes since it can work as a second messenger to facilitate the proliferation of cells, whereas excessive ROS accumulation is toxic, forcing cells to endure harmful oxidative stress conditions. Besides that, the type and the subcellular location of ROS are also essential for maintaining cellular processes. In general, cancer cells lie in increased ROS levels due to over-activated oxygen metabolism. As a consequence, these cells develop sophisticated anti-oxidant systems to maintain ROS levels at optimal concentrations (6). Considerable evidence has shown that ROS dysregulation activates oncogenic pathways and promotes phenotypic transformation to a malignant state (7). However, current ROS metabolic studies are faced with dilemmas: why ROS-generating or ROS-scavenging processes are both deemed oncogenic events (8, 9). Therefore, the exploration of ROS status across cancer types is urgently required to clarify ROS metabolic equilibrium in tumor biology.

ROS plays an important role in modulating treatment outcome. On one hand, conventional anti-tumor therapies have used ROS (such as hydroxyl radicals or singlet oxygen) as common mechanisms to damage tumor cells (10, 11). The activation of ROS-scavenging systems eliminates these overloaded radicals, leading to therapy resistance. On the other hand, ROS, as a second messenger regulating oncogenic signaling like NF-KB and PI3K-AKT, abolishes or reduces pathway-targeting regimens (12). Therefore, ROS modulation is a promising strategy to improve the therapeutic efficacy of established treatments (13, 14). However, ROS modulation strategy only achieved limited advances (7, 15) and even led to opposite clinical outcomes in different cancer types (16, 17). This situation may be due to a lack of methods to comprehensively evaluate ROS status (18). Currently, ROS metabolism is primarily assessed by fluorescence probes reacting directly with ROS or by quantifying the expression of key ROS metabolic enzymes (19, 20). However, these two measures show limitations in clinical use due to methodological reasons. By using fluorescence probe method, tumor cells need to be separated and prepared into single cell suspension, while tumor is characterized as an abnormal organ that ROS metabolic status of tumor cell suspension ignore the widespread role of tumor microenvironment. Additionally, *ex vivo* preparation including cell separation, cultivation, and incubation for several hours dramatically alters ROS status *in vivo*. On the other hand, detecting the expression of key enzyme is limited due to only revealing specific steps of ROS metabolism. However, ROS metabolism is a cascade process with complex generating and scavenging interactions that the state of a step cannot fully represent the entire equilibrium exactly. Therefore, novel methodologies and approaches are required to evaluate ROS status in cancer management.

In this study, we investigated The Cancer Genome Atlas (TCGA) multi-omics data (21) to comprehensively explore ROS metabolism across 22 human cancers. We developed a transcriptomic-based method to depict ROS metabolism in five dimensions (accumulation, oxidative stress, scavenging, biosynthesis, and subcellular sources), which provides several advantages over traditional testing methods. Cancer patients were classified into eight clusters, based on our ROS indexes, with various clinical and molecular features. We also observed that anti-tumor drug efficacy was predicted by our indexes; furthermore, we demonstrated that treatment efficacy was improved by modulating ROS metabolism. Taken together, this study provides a methodological framework to understand ROS metabolism across various cancer types. Importantly, the study provides insightful perspectives on ROS clinical evaluations and translational medicine.

## Materials and Methods

### Data Sources

mRNA, somatic mutations, and clinical data from 22 solid tumors were downloaded from the TCGA data portal (https://portal.gdc.cancer.gov/), while miRNA, protein expression, and copy number variation (CNV) were downloaded from the UCSC Xena website (https://xenabrowser.net). Drug sensitivity area under the dose–response curve (AUC), IC50, and gene expression profiles for treated cell lines were downloaded from the Genomics of Drug Sensitivity in Cancer (GDSC) website (http://www.cancerrxgene.org/downloads) (22). Gene signatures of biological processes (C5.BP.v6.1) were downloaded from the Molecular Signatures Database (http://software.broadinstitute.org/gsea/index.jsp) (23).

### Bioinformatic Analysis

Bioinformatic analysis was done as follows with R package or online websites. The code can be acquired from the corresponding author upon request.

### Gene Set Interaction Analysis

To find out the interactions between different gene sets, Amigo2 was used to explore the interactions between different gene sets (amigo.geneontology.org/) by drawing directed acylic graph. GO ID was used as input for Amigo2 analysis, and the result was visualized with Cytoscape.

### GSVA Analysis

To evaluate the gene set expression among different patients or different CCLE cell lines, GSVA R package was used (24). RNA-seq expression profile and gene signature were used as the input.

### Calculating the Geometric Mean Evaluation of Gene Sets

Geometric mean of gene sets was acquired by calculating the geometric mean of the gene expression that consists this gene set.

### Evaluation of ROS Metabolism by a Five-Dimensional Index

A five-dimensional index was put forward by combining the ROS-metabolism-related gene sets acquired from MsigDB database with proper operation. The geometric mean of each gene set was chosen for further operation. Details about the five indexes can be found in Supplementary Table 3.

### Reclassification of the TCGA Samples

Reclassification of the TCG samples was done by using the hierarchical clustering method provided by pheatmap R package. The five ROS indexes of each TCGA sample were used as clustering features, and eight clusters were acquired.

### T-SNE Analysis

T-SNE package of R was used to profile ROS metabolism patterns to see whether ROS indices can be used to distinguish different samples. The five ROS indexes of each TCGA sample were used as inputs for T-SNE analysis.

### Tumor Purity, Immune Score, and Stromal Score Calculation

Microenvironment composition parameters including tumor purity, immune score, and stromal score were calculated as previously described using the estimate method (25).

### Protein–Protein Interaction Analysis

The protein–protein interaction analysis was done by the String website (https://string-db.org), and the protein with a degree larger than 1.5-fold of the average degree of the input genes was defined as the hub gene.

### Definition of Drug Sensitivity and Drug Resistance

The classification of being drug-sensitive or drug-resistant was defined according to GDSC's work (26). Briefly, if the IC50 of a certain drug was higher than the maximum screening concentration, the drug was defined as chemotherapeutically resistant; otherwise, the drug was defined as chemotherapeutically sensitive. The maximum screening concentration is determined using clinical data indicating peak plasma concentrations in human projects.

### The Relationship Between Drug Sensitivity Data and ROS Indexes

The relationship between drug sensitivity data and ROS indexes was done by the following steps: (1) the expression of ROS indexes among different cell lines was calculated in the same way as the TCGA samples by using the expression profile of different cell lines provided in the GDSC website and (2) calculating the Spearman correlation between ROS indexes and IC50 data.

### Outlier Filtering

After the scores of each index were calculated, outliers indicated by R were removed. In general, approximately the top and the bottom 5% of samples of each index was removed to filter outliers.

### Cell Culture

Human glioma cell line U87, human glioma cell line LN229, human glioma cell line U251, human acute myeloid leukemia cell line THP-1, and human renal carcinoma cell line ACHN were purchased from the Chinese Academy of Sciences cell bank (Shanghai, China). The U87, LN229, and U251 cells were maintained in Dulbecco's Modified Eagle's medium (HyClone, Logan, UT, USA). The THP-1 cells were maintained in Roswell Park Memorial Institute-1640 medium (HyClone), and the ACHN cells were maintained in Minimum Eagle's Medium (HyClone). All cell lines were supplemented with 10% fetal bovine serum (HyClone) and 1% penicillin/streptomycin (Gibco, Carlsbad, CA, USA) at 37°C in 5% CO_2_. Hydrogen peroxide (H_2_O_2_) was added according to experimental requirements, but generally, 1–20 μM was considered a low concentration, while 10–100 μM was considered a high concentration according to the specific cell line chosen and the specific experiment conducted.

### Western Blotting

Cells were lysed in RIPA lysis buffer for 30 min and centrifuged at 12,000 rpm for 15 min at 4°C. The supernatant was removed, and protein concentration was measured by the BCA protein assay method according to the manufacturer's protocol (Beyotime, Shanghai, China). Then, 20 ug of protein from each sample was separated by electrophoresis, transferred to polyvinylidene fluoride membranes (Millipore, MA, USA), and blocked in 5% skimmed milk for 1 h at room temperature. Primary antibodies (p-NF-KB, Cell Signaling Technology, Boston, USA; NF-KB, Cell Signaling Technology, Boston, USA; p-ERK, Cell Signaling Technology, Boston, USA; ERK, Cell Signaling Technology, Boston, USA; p-AKT, Cell Signaling Technology, Boston, USA; AKT, Cell Signaling Technology, Boston, USA; p-STAT3, Cell Signaling Technology, Boston, USA; STAT3, Cell Signaling Technology, Boston, USA; and GAPDH, Proteintech, Wuhan, Hubei, China) were incubated with membranes overnight. On the following day, secondary antibodies (peroxidase-conjugated affinipure goat anti-rabbit IgG or anti-mouse IgG, Proteintech, Wuhan, Hubei, China) were used at room temperature for 1 h. Protein bands were detected using chemi-luminescence ECL reagents (Tanon, Shanghai, China).

### Immuno-histochemical Staining

Immuno-histochemical staining was performed as previously described (1). Briefly, sections were hybridized with a primary antibody (Ki-67, Servicebio, Wuhan, Hubei, China) at 4°C overnight and, on the next day, hybridized with a secondary antibody (goat anti-rabbit, Servicebio, Wuhan, Hubei, China) at 37°C for 30 min. After washing in phosphate-buffered saline, sections were stained with DAB (Servicebio, Wuhan, Hubei, China) for 3 min, rinsed in water, and co-stained with hematoxylin.

### Detection of ROS *in vitro*

Cells were plated in 96-well plates (five replicates per condition, at 2.5 × 10^3^ cells per well) and incubated overnight at 37°C in 5% CO_2_ before detecting. Total amount of ROS was detected with (DCFH-DA); mitochondrial ROS was detected with MitoSOX. After the cells have adhered to the plate, these were pretreated with 150 uM of Trig for 24 h to detect the biosynthetic ability of ROS or pretreated with 50 uM of H_2_O_2_ for 6 h to detect the scavenging ability of ROS.

### Cell Proliferation Assay

The effects of drugs with different ROS levels on cell proliferation were determined by the CellTiter 96 Aqueous One Solution Cell Proliferation Assay Kit (Promega, Madison, WI, USA) according to the manufacturer's instructions. Cells were plated in 96-well plates (three replicates per condition at 5 × 10^3^ cells per well) and incubated overnight at 37°C in 5% CO_2_. On the following day, the cells were treated with different concentrations of H_2_O_2_ or N-acetyl-L-cysteine (NAC) with selected drugs simultaneously. In detail, 5 μM of H_2_O_2_ was used to perform as second messengers, while 15 μM of H_2_O_2_ was used to bring cells into oxidative stress; NAC was added with a concentration of 2.5 mM; bortezomib was used with a concentration of 10 nM with H_2_O_2_ or with a concentration of 5 nM with NAC in the U87 cell line, with a concentration of 5 nM with H_2_O_2_ or with a concentration of 2.5 nM with NAC in LN229 cell line and with a concentration of 20 nM with H_2_O_2_ or with a concentration of 10 nM with NAC in U251 cell line; docetaxel was used with a concentration of 40 nM; methotrexate was used with a concentration of 8 μM; and vorinostat was used with a concentration of 5 μM. Then, 20 μl of CellTiter 96 Aqueous One Solution reagent was added directly to the wells, incubating for 2 h and recording the absorbance at 490 nm on an EnVision Plate Reader (Perkin Elmer, Waltham, MA, USA) at 0, 24, 48, and 72 h post-drug administration. Data analysis was performed using Prism version 7.00 (Graph Pad software). Unpaired Student's *t*-test was applied as the statistical method.

### *In vivo* Xenograft Drug Sensitivity Assay

Twenty athymic female BALB/c nude mice (5 weeks old) were obtained from Beijing Vital River Laboratory Animal Technology Co., Ltd. (Beijing, China). Approximately 2 × 10^6^ U87 cells were transplanted into the right flank of the animals in inoculation volumes of 200 μl sterile media. At day 7 after implantation, the animals were randomly assigned to one of four groups with different purposes. From that time on, bortezomib and H_2_O_2_ were administrated as follows before the animals were sacrificed. Bortezomib (MCE, NJ, USA) was administered (0.40 mg/kg) i.p. every 3 days after the animals were assigned to different groups. H_2_O_2_ was received 1 h after bortezomib administration by intra-tumoral injection, with a concentration of 5 μM, and the injection volume is 10% of the tumor volume. The tumor volume was calculated by length × width^2^/2. Unpaired Student's *t*-test was applied as the statistical method.

### Statistical Analyses

All statistical analyses were performed using R 3.5.1 (https://www.r-project.org), SPSS 12 (http://www.spss.com), or Prism 7.0 (https://www.graphpad.com/scientific-software/prism/). Prognostic values of gene sets or indices were analyzed by univariate Cox regression analysis. Patient survival in different clusters was analyzed using log-rank and Kaplan–Meier analyses. Unpaired comparison using chi-square test was used to find the characteristic mutations in each cluster. Unpaired comparison using Student's *t*-test was used to find the altered miRNA or gene ontology in each cluster. CNV with frequency larger than 10% was defined as characteristic CNV. Unpaired Student's *t*-test was used to analyze the difference of drug efficacy between the high-index group and the low-index group. A two-tailed *p* < 0.05 was accepted as statistically significant. Multiple testing correction using Bonferroni method was conducted in necessary situations, and false discovery rate (FDR) < 0.05 was defined as the statistical threshold.

## Results

### A Five-Dimensional Index That Reflects ROS Metabolic Status

**Figure 1:**
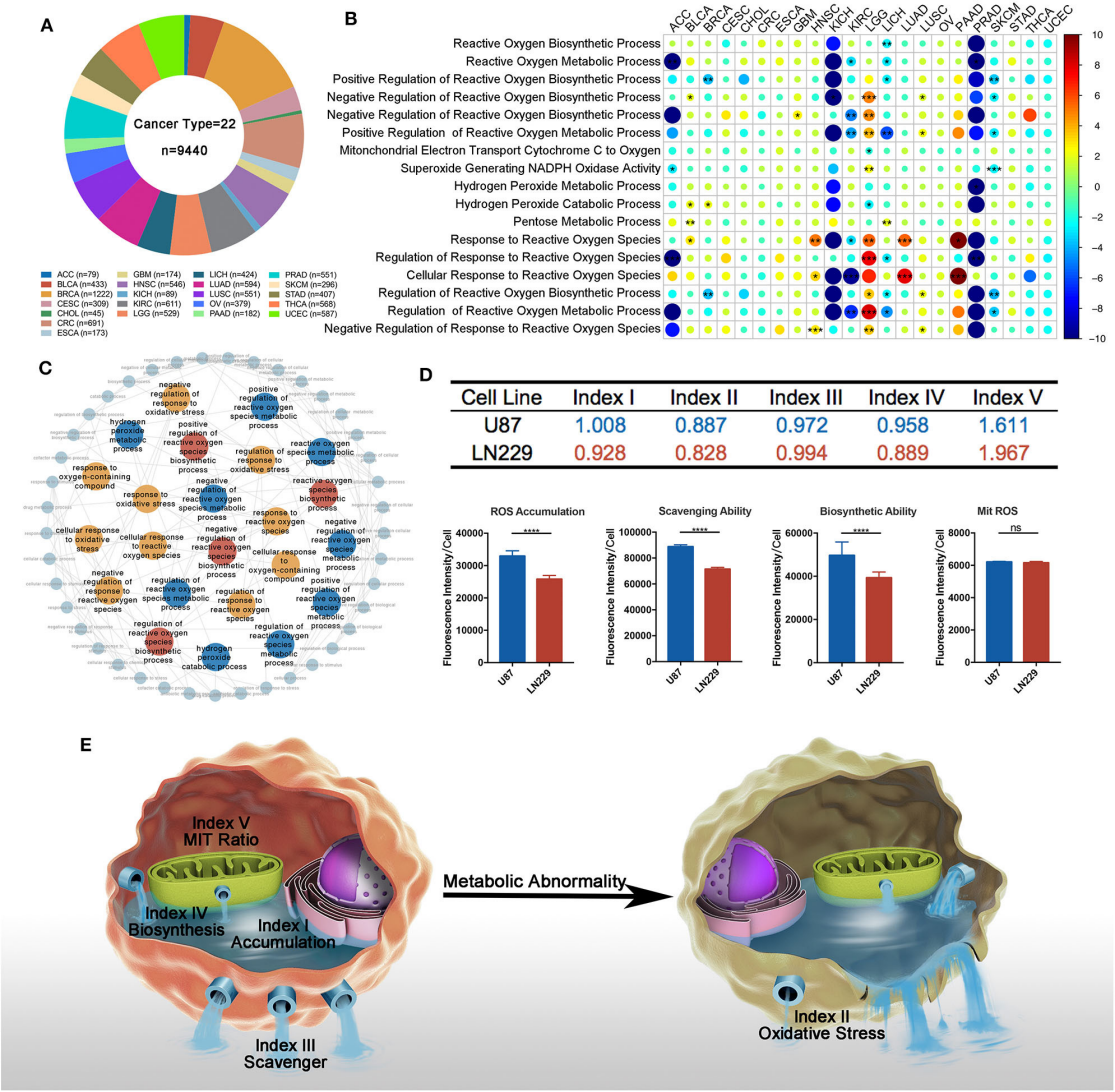
Overview of reactive oxygen species (ROS) metabolism across different cancer types. **(A)** Sample composition in this study (cancer types = 22, *n* = 9,440). Sample number varied from 45 in CHOL to 1,222 in BRCA. **(B)** Univariate cox results of each ROS metabolism-related gene set score in different cancer types. All ROS metabolic processes show prognostic values at least in one cancer type; 15 cancer types were determined at least by one ROS process; 13 processes exerted divergent prognostic roles in different cancer types. red = risk factor, blue = protective factor; *p < 0.05, **p < 0.01, ***p < 0.001; hazard ratio (HR) lesser than 1 is transformed into (-1) * 1/HR. **(C)** Interactions between ROS-metabolism-related gene signatures and other related signatures: red = biosynthetic related signatures, blue = metabolic-related signatures, yellow = response-related signatures. **(D)** Computational comparison and experimental comparison of ROS metabolism between U87 and LN229. U87 showed a higher ROS accumulation, a stronger ROS scavenging ability, a stronger ROS generating ability, and a severer ROS stress condition compared to LN229. ****p < 0.0001. **(E)** Overview of ROS-metabolism-related indexes: index I, ROS accumulation; index II, oxidative stress; index III, ROS scavenging ability; index IV, ROS biosynthetic ability; and index V, subcellular ROS origins.

To explore ROS metabolic processes in different cancer types, multi-omics data were firstly downloaded from the TCGA website and Xena website (Figure 1A). We then summarized 17 established gene sets associated with ROS metabolism from the Msigdb database (Supplementary Table 1), and GSVA was used to qualify a gene set's activities. Since survival was the most important factor in cancer therapy, we firstly explore the prognostic value of the ROS-related gene sets. The univariate Cox model was conducted to test their prognostic values. We found that all ROS metabolic processes (100.0%) show prognostic values at least in one cancer type, and the prognostic status of 15 cancer types was determined at least by one ROS process (Figure 1B, Supplementary Table 2; for visualization, HR < 1 was transformed into (-1)/HR). Thirteen processes exerted divergent prognostic roles in different cancer types, *e*. *g*., the positive regulation of ROS metabolism was a risk factor in lower-grade glioma (LGG) but served as a protective factor in kidney renal clear cell carcinoma (KIRC). LGG was also highlighted as a cancer type whose outcome was determined by most ROS metabolic processes (13/17, 76.5%).

Since most ROS metabolic processes are characterized by a cascade of reactions, we next sought to depict the interplay of different ROS metabolic processes. Using Amigo2, we drew the directed acylic graph of these gene sets. According to the interactions between the ROS gene sets, the ROS metabolic processes could be classified into three groups, including (1) ROS biosynthesis, (2) ROS scavenging, and (3) ROS-mediated cellular stress (Figure 1C). These three groups contained a modest overlap, such that eight and five common genes were identified among them and their regulatory processes, respectively (Supplementary Figures 1A,B). These results indicated that ROS metabolism is a complex cascade process.

To comprehensively depict this complex cascade process, we developed a transcriptome-based approach containing five indexes to depict the landscape of ROS metabolism based on one study. This study measured several coefficients and put forward several equations in the cascade of ROS metabolism (27). Index I reflected ROS accumulation, index II reflected oxidative stress levels, index III indicated ROS scavenging abilities, index IV cataloged ROS biosynthetic ability, and index V represented ROS subcellular sources (mitochondria/cytoplasm; a higher index V value indicated more ROS generated from the mitochondria as biological byproducts). Detailed calculating formula can be found in Supplementary Table 3.

To verify the reliability of our indexes, we calculated the value of each index in well-established tumor cell lines (Supplementary Table 4). We selected two well-established glioblastoma multiforme (GBM) cell lines (LN229 and U87) for *in vitro* experiments to validate the bioinformatics result. In comparison with LN229, U87 possessed higher indexes I and IV and lower indexes II, III, and V (Figure 1D). DCFH-DA was used to compare the ROS accumulation (index I) between these two cell lines, showing that U87 was enriched with ROS than LN229. To test the ROS scavenging ability (index III) between these two cell lines, exogenous H_2_O_2_ was administered 2 h before DCFH-DA application. Less ROS remained in LN229 medium than in U87 medium, which indicated that LN229 was featured by a powerful ROS scavenging ability. To compare the ROS biosynthesis ability (index IV) between these two cell lines, cells were pretreated with Trig for 24 h to block the ROS scavenging process, and U87 showed higher ROS biosynthetic ability than LN299. Then, Mitosox was used to detect mitochondrial ROS accumulation. We found that U87 and LN229 had similar mitochondria ROS accumulation. Considering the lower ROS accumulation in LN229 than in U87, LN229 displayed a higher mitochondria/cytoplasmic ratio (index V) (Figure 1D). Therefore, the ROS computational results were validated by experiments, indicating that our method was competent to precisely evaluate ROS metabolic status (Figure 1E). Then, we used our indexes to evaluate ROS metabolism in TCGA samples. After calculating and filtering the outliers indicated by R software, 7,559 samples in TCGA across 22 cancer types were included for analysis (Supplementary Figure 1C).

Finally, we performed correlation analyses to explore the interactions between each index using TCGA samples (Supplementary Table 5). Index I was highly associated with index IV (*r* = 0.81, *p* < 0.0001), indicating that cellular ROS accumulation was predominantly determined by biosynthetic ability. Besides that, index III and index IV exhibited no correlations (*r* = −0.005, *p* = 0.9822), suggesting that ROS scavenging and ROS biosynthetic abilities were two independent processes.

### Characterization of ROS Indexes Across Different Cancer Types

**Figure 2:**
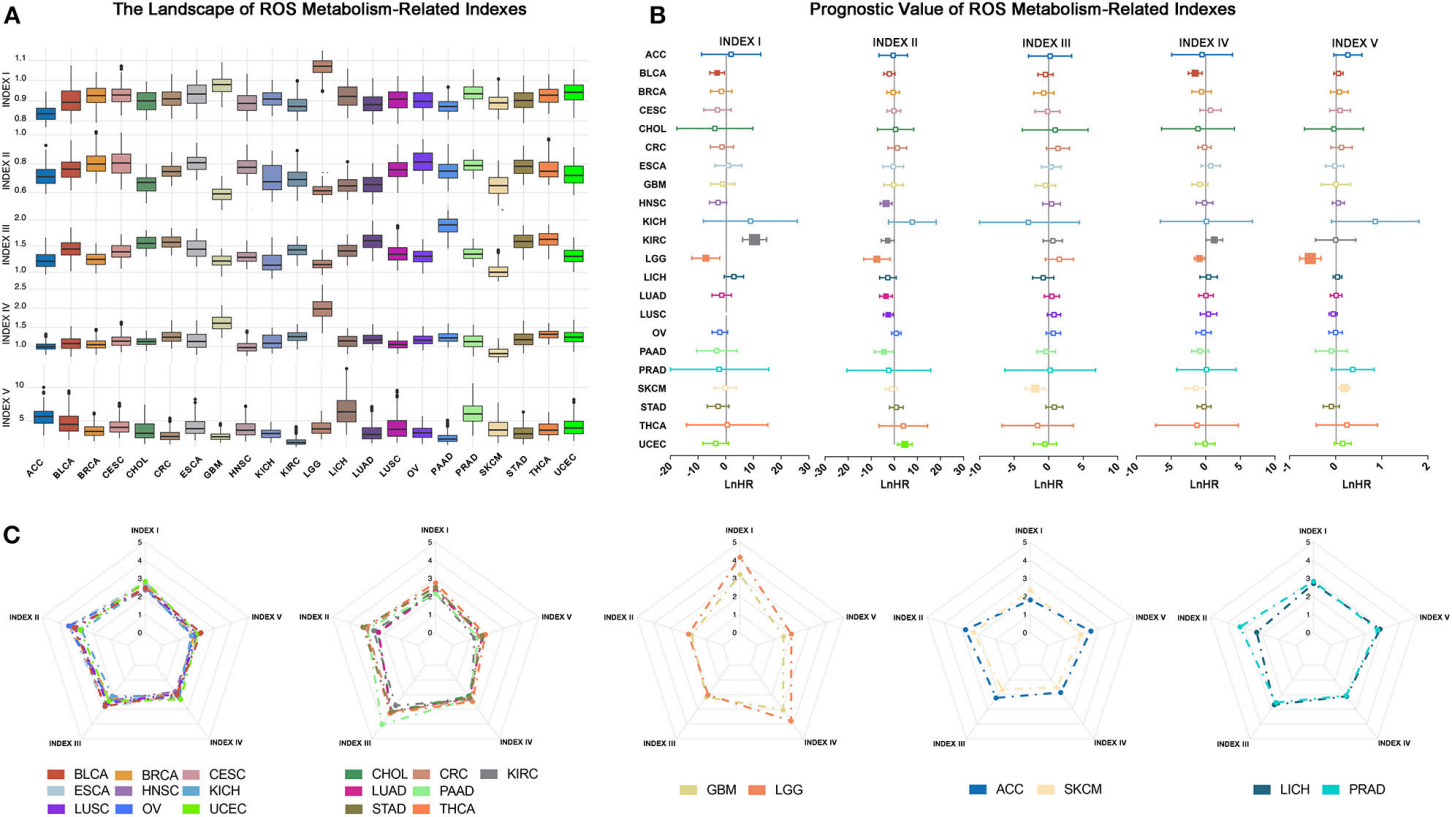
Overview of reactive oxygen species (ROS) indexes across different cancer types. **(A)** The distribution of ROS indexes across different cancer types. Glioma, including lower-grade glioma (LGG) and glioblastoma multiforme (GBM), had the highest level of ROS accumulation and ROS biosynthetic ability, while adrenocortical carcinoma or skin cutaneous melanoma exhibited lower profiles of most ROS indexes. **(B)** Prognostic value of ROS indexes across different cancer types: positive, risk factor; negative, protective factor; solid square, with significance; the square size is negatively correlated with *p*-value. The hazard ratio is Ln-transformed. **(C)** Five groups of ROS metabolic patterns: from left to right, group 1 to group 5. Group 1, which mainly consisted of squamous and gynecological carcinomas, showed elevated oxidative stress. Group 2, which mainly contained adenocarcinomas, was characterized by increased ROS scavenging abilities. Group 3 constituted tumors of the central nervous system (GBM and LGG) and showed high levels of ROS accumulation and generation potential. Groups 4 and 5 were characterized by low and moderate ROS metabolic patterns, respectively.

We firstly depicted index distributions to investigate ROS metabolic status across different cancers (Figure 2A). We observed distinct ROS metabolic profiles in several cancer types. Gliomas, including GBM and LGG, had the highest ROS accumulation, which could be explained by its strongest ROS biosynthesis but relatively weakened scavenging ability. We also observed that cancers such as adrenocortical carcinoma (ACC) and skin cutaneous melanoma exhibited lower profiles of most ROS indexes, suggesting a relative lack of ROS metabolism in these cancers.

We then explored the prognostic influences of ROS indexes in cancers and observed different prognostic effects of ROS indexes among cancer types (Figure 2B; Supplementary Table 6). For cancers such as KIRC and LGG, most ROS indexes (four out of five in LGG and three out of five in KIRC) had prognostic values. However, for ACC and breast invasive carcinoma (BRCA), no indexes were statistically significant. Index II was highlighted to correlate with overall survival in most cancer types (*N* = 7), indicating the prognostic importance of oxidative stress in human cancers.

Finally, we assessed similarities and differences of ROS metabolism in different cancer types. The arithmetic averages of each index were used to represent the average ROS metabolism in each cancer type. By using hierarchical clustering to the arithmetic averages, cancer types were classified into five groups based on ROS metabolic status (Figure 2C and Supplementary Figure 2A). Group 1, which mainly consisted of squamous and gynecological carcinomas, showed elevated oxidative stress. Group 2, which mainly contained adenocarcinomas, was characterized by increased ROS scavenging abilities. Group 3 constituted tumors of the central nervous system (GBM and LGG) and showed high levels of ROS accumulation and generation potential. Groups 4 and 5 were characterized by low and moderate ROS metabolic patterns, respectively. These results suggest different distributions and clinical features of ROS metabolism in human cancers.

### Tumors Are Classified Into Eight Clusters According to ROS Indexes

**Figure 3:**
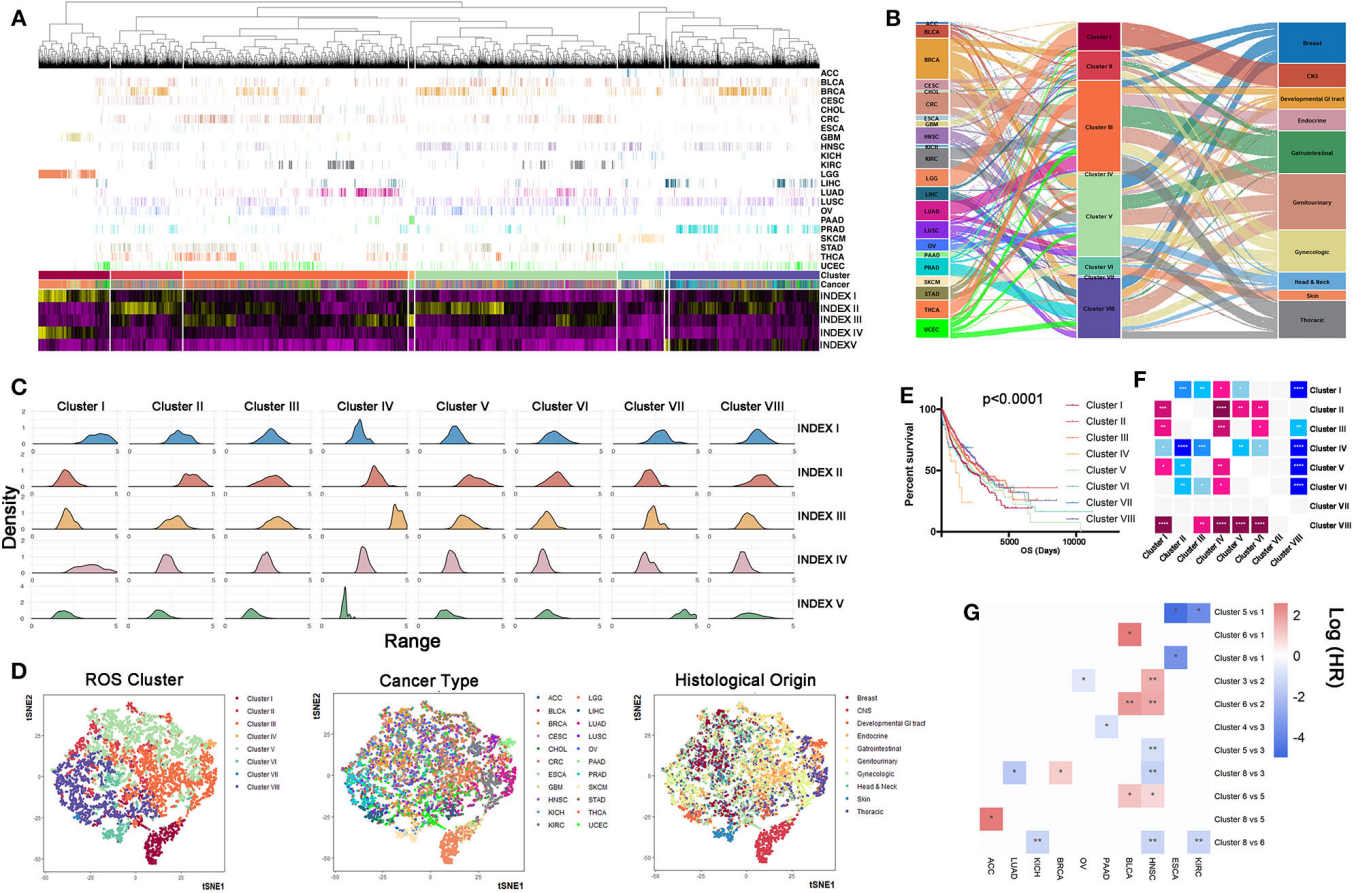
Samples can be reclassified into different clusters according reactive oxygen species (ROS) indexes. **(A)** Clustering analyses of samples based on ROS indexes: from left to right, cluster I to cluster VIII. Cluster III had the largest number of samples, while cluster VII had the lowest number of samples. **(B)** Distribution density of each index in each cluster: from left to right, cluster I to cluster VIII. Cluster I was characterized by high ROS levels and enhanced biosynthetic processes. Cluster II exhibited elevated oxidative stress levels. Cluster IV was characterized by a reinforced ROS scavenging phenotype. Cluster VII tumors were associated with ROS generated from the mitochondria. **(C)** The composition of cancer types and histological origins in each cluster: left, cancer type; middle, clustering cluster; right, histological origin. Clusters I, IV, and VII were relatively homogenous, whereas other clusters were heterogeneous. **(D)** t-SNE based reducing dimension analysis using ROS indexes, cancer types, or histological origins to separate cancer samples. ROS indexes could be used to reclassify The Cancer Genome Atlas samples. **(E)** Survival curves of clusters (I–VIII), using the log-rank method. Clusters I and IV had significantly reduced survival times, while cluster II and VIII had significantly prolonged survival times. **(F)** Comparison of prognosis differences between each cluster: *p < 0.05, **p < 0.01, ***p < 0.001, ****p < 0.0001. Horizontal direction: blue = poor prognosis, pink = good prognosis. Vertical direction: pink = poor prognosis, blue = good prognosis. **(G)** Survival comparison of cancers between different clusters: *p < 0.05, **p < 0.01.

To discriminate tumor samples according to ROS metabolic status, hierarchical clustering was used to classify all tumor samples, regardless of their histological types. Patients were classified into eight clusters according to ROS indexes (Figure 3A, Supplementary Table 7). Density curves suggested that eight ROS clusters owned distinct ROS metabolic phenotypes (Figure 3B). Cluster I was characterized by high ROS levels and enhanced biosynthetic processes. Cluster II exhibited elevated oxidative stress levels. Cluster IV was characterized by a reinforced ROS scavenging phenotype. Cluster VII tumors were associated with ROS generated from the mitochondria. We next analyzed tumor composition for each cluster. Clusters I, IV, and VII were relatively homogenous, whereas the other clusters were heterogeneous (Figure 3C). Clusters I, IV, and VII mainly consisted of gliomas (79.3%), pancreatic adenocarcinoma (98%), and liver hepatocellular carcinoma (96.9%), respectively. Importantly, almost all (95.8%) glioma (GBM and LGG) patients were grouped into cluster I, while other tumor types were divided into several clusters. Further t-SNE analyses showed that ROS metabolism provided a novel perspective to reclassify tumors, which is independent of their pathological diagnosis or histological origin (Figure 3D). With regards to prognostic implications, patients in different ROS clusters experienced different clinical outcomes (Figures 3E,F, *p* < 0.0001). Clusters I and IV had significantly reduced survival times, while cluster II and VIII had significantly prolonged survival times. To eliminate the effect of cancer type in determining a patient's survival, we also calculate the hazard ratio (HR) of different cancer types in different clusters, and the result is consistent with the pan-cancer survival analysis. For ESCA and KIRC, patients that were classified in cluster I have shown a reduced survival time than in other clusters; for HNSC and BLCA, patients in cluster II had a prolonged survival time than in other clusters, while for LUAD and HNSC, patients who fell in cluster VIII had a prolonged survival time compared to those in other clusters (Figure 3G). Taken together, our ROS indexes reclassified tumor samples into eight ROS clusters with important clinical implications.

### Molecular and Biological Landscapes in ROS Clusters

**Figure 4:**
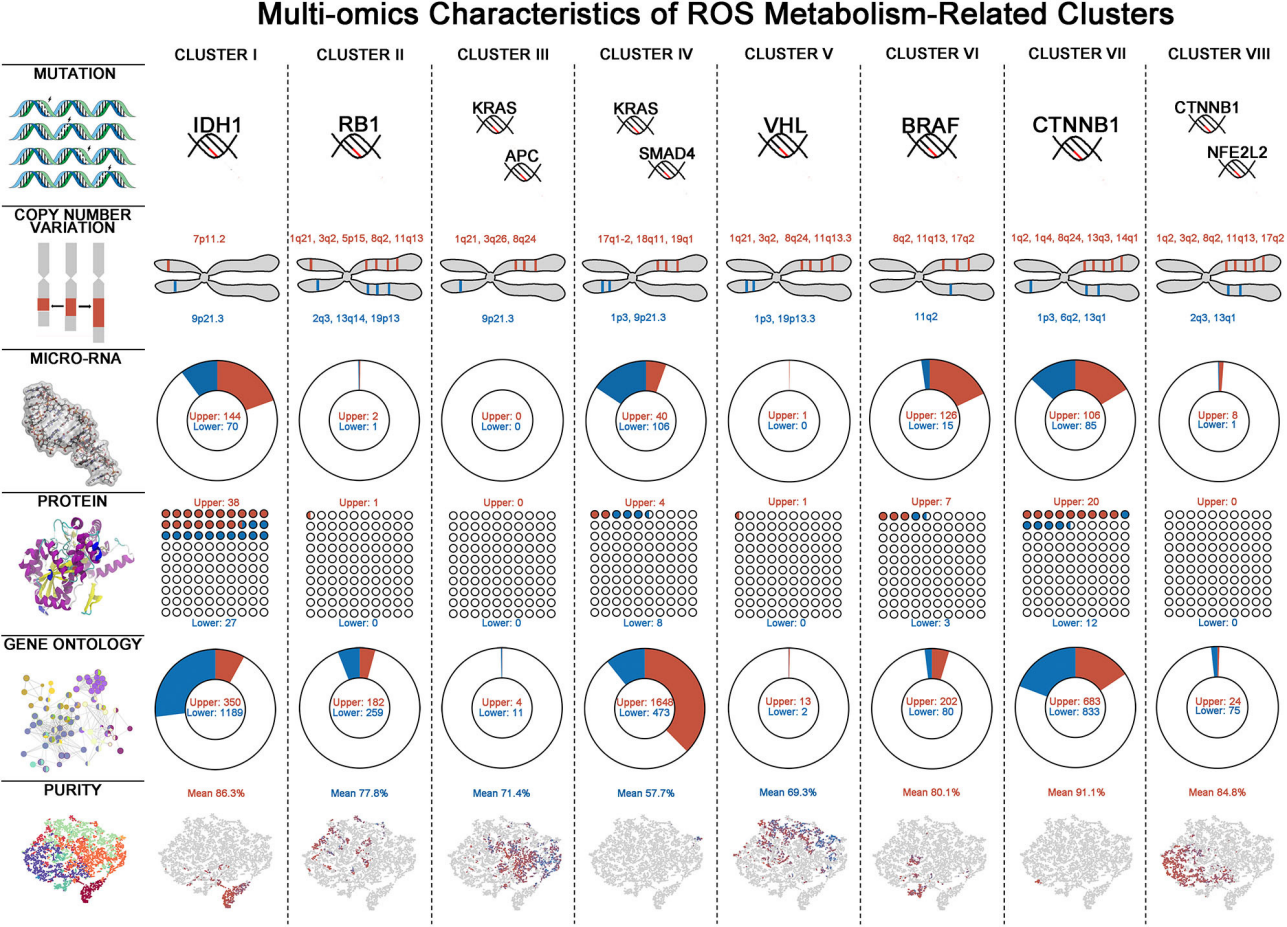
Multi-omics characteristics of eight reactive oxygen species (ROS) clusters. Multi-omics characteristics including mutation, copy number variation (CNV), micro-RNA, protein, Gene Ontology (GO), and tumor purity alterations in each ROS cluster. For mutations, characteristic mutations with most occurrences are shown—for CNV: red = amplification, blue = deletion; for micro-RNA, protein, and GO: red = up-regulated, blue = down-regulated; and for tumor purity: red = high purity, blue = low purity.

To gain some insights of ROS metabolism from a multi-omics perspective, we next integrated mutations, CNVs, miRNAs, and RPPA data to identify key multi-omics features driving different ROS metabolism (Figure 4, Supplementary Table 8). Considering mutations, cluster VI has the largest number and the highest frequency of mutations, while cluster IV was just characterized by mutations on KRAS and SMAD4. Cluster I was characterized by enrichment of mutations in IDH1 (52%, FDR = 1.42 × 10^−8^) and ATRX (27%, FDR = 0.0232), consistent with their predominance in glioma samples (28). As for CNVs, several chromosomal loci were identified with various amplification or deletion frequencies among ROS clusters. Two important tumor-suppressor genes, CDKN2A (chromosome 9p21.3) and CDKN2B (chromosome 9p21.3), were deleted in all clusters except for II. In cluster I, the EGFR locus (chromosome 7p11.2, 14.3%) was significantly amplified, consistent with EGFR chromosomal amplification during glioma progression (29). miRNA expression also varied in different ROS clusters. Featured up-regulated miRNA varied from 144 in cluster I to 0 in cluster III, while down-regulated RNA varied from 106 in cluster IV to 0 in cluster III and cluster V. Interestingly, we observed that featured miRNAs activating (miR-128, FDR = 2.78 × 10^−3^) or suppressing (miR-146, FDR = 2.95 × 10^−3^ and miR-27, FDR = 1.33 × 10^−11^) NF-κB were, respectively, up- or down-regulated in cluster I. Furthermore, reverse-phase protein array-based analyses provided important molecular clues in different clusters. Mtor (FDR = 3.18 × 10^−3^) and p-Mtor (FDR = 2.62 × 10^−3^) were found to be up-regulated in cluster I, which was consistent with the highest ROS accumulation level in this cluster. Besides that, p-AKT (FDR = 1.04 × 10^−4^) was up-regulated in cluster I, which could further accelerate the phosphorylation of NF-KB. In cluster IV, increased ROS scavenging phenotypes may be derived from over-phosphorylated AMPK (FDR = 1.23 × 10^−4^), which is critical for activating antioxidant pathways (30). The multi-omics alterations would lead to functional changes of tumor samples and functional enrichment analysis, indicating that homogenous clusters tended to have more characteristic GO alterations than heterogeneous clusters. Consistent with miRNA profiling, NF-κB negative-regulation-related GOs (Go_Negative_Regulation_of_Nf_Kappab_Transcription_Factor_Activity, FDR=9.67 × 10^−6^ and Go_Negative_Regulation_of_I_Kappab_Kinase_Nf_Kappab_Signaling, FDR = 1.96 × 10^−4^) were down-regulated in cluster I. Taken together, these results provide an overview of the multi-omics differences associated with ROS metabolism.

Recent research has emphasized the role of the tumor microenvironment in tumor biology, as there appears to be complex mutual interplay between ROS expression and microenvironment remodeling (15). Microenvironment parameters, including tumor purity and immune and stromal scores (25), were calculated to explore the microenvironment composition in ROS clusters. The positive relationship between index I and tumor purity indicated that tumor cells predominantly contributed to ROS accumulation (Supplementary Figures 2B–D). Strong correlations were observed between index V and microenvironment composition parameters, further suggesting that mitochondria-derived ROS was predominantly responsible for tumor microenvironment establishment (Supplementary Figures 2B–D). We next assessed the cellular proportions for each ROS cluster. As expected, microenvironment composition varied among ROS clusters. Cluster IV had the lowest enrichment of tumor cells, coupled with the most abundant leukocyte and stromal cell levels (Supplementary Figure 3A). Prognostic analyses indicated that tumor purity, immune score, and stromal score played important roles in determining clinical outcomes in cluster I (Supplementary Figures 3B–D), which was consistent with our previous study (25). Additionally, immune cells and stromal cells were risk indicators for cluster III, whereas tumor cell proportions had limited prognostic implications (Supplementary Figures 3B–D). These results together indicated that ROS metabolism modulated cellular composition and determined the clinical significance of tumor microenvironments.

### The Impact of ROS Metabolism on Drug Responses

**Figure 5:**
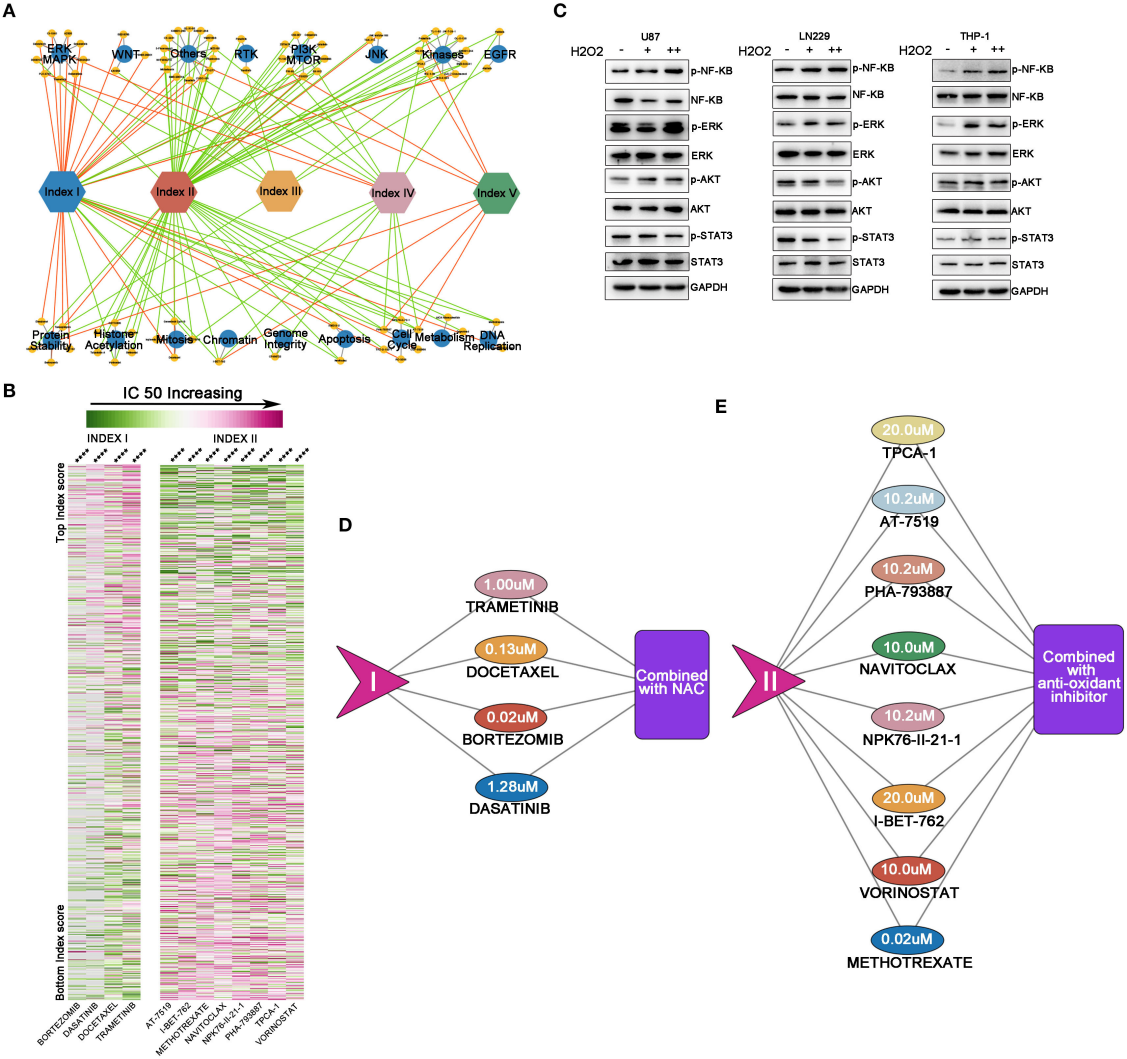
Reactive oxygen species (ROS)-based drug efficacy evaluation network. **(A)** Associations between index values and drug sensitivities across 1,066 cancer cell lines, using Spearman's rank correlation. Indexes I and II were important nodes for the network. Increasing index I mainly positively correlated with the IC50 of drugs targeting kinases on oncogenic pathways, while index II predominantly sensitized tumor cells to drugs targeting the PI3K/mTOR pathway, kinases, chromosome modifications, and the cell cycle. Red line = positive correlation, green line = negative correlation. Only drugs with moderate correlations are shown: yellow circle = drugs, blue circle = drug targets. **(B)** Heat map showing drugs with significant correlations with indexes I and II. The left panel shows index I and correlated drugs. The right panel shows index II and correlated drugs. With ROS accumulation increasing, the IC50 of four drugs correlated with index I increased; with oxidative stress degree deepening, normal cell cycle or DNA modification process would be affected and decrease the IC50 of the drugs correlated with index II. From green to pink, the IC50 increases; ****p < 0.0001. **(C)** By supplementing cells with exogenous H_2_O_2_, NF-κB and ERK were activated in these three cell lines; the STAT3 pathway was inhibited in glioma U87 and LN229 cell lines while activated in the THP-1 cell, whereas the AKT pathway was activated in U87 and THP-1 cell lines but inhibited in LN229 cell line. **(D,E)** The maximum concentration tolerance in the body of drugs from **(B)** and potential synergic strategies to decrease drug IC50. For drugs correlated with index I, N-acetyl-L-cysteine should be combined to increase drug efficacy, while for drugs correlated with index II, anti-oxidant inhibitor should be combined to increase drug efficacy.

Since ROS metabolic phenotypes were distinct among the above-mentioned clusters, we sought to explore whether various ROS status determined the therapeutic efficacy of anti-tumor drugs. We reviewed information from 251 anticancer drugs applied to 1,066 cancer cell lines (Genomics of Drug Sensitivity in Cancer) (22) by calculating the index value and drug AUC. We found that 81.3% of drugs (204/251) correlated with our ROS indexes (Supplementary Table 9). The correlation between drug AUC and indexes was used to establish a drug efficacy evaluation network (Figure 5A). Our results indicated that indexes I and II were important nodes for the network, highlighting their roles in regulating drug efficacy. Notably, increasing index I mainly positively correlated with the IC50 of drugs targeting kinases on oncogenic pathways, while index II predominantly sensitized tumor cells to drugs targeting the PI3K/mTOR pathway, kinases, chromosome modifications, and the cell cycle.

Four out of five drugs with highest correlations to index I were signaling or kinase inhibitors [dasatinib inhibits STAT3 pathway (31); bortezomib inhibits NF-KB pathway (32); docetaxel inhibits PI3K-AKT pathway (33), while trametinib inhibits MEK-ERK pathway (34)]. These key pathways are important in cellular survival, proliferation, or angiogenesis in tumor biology. With ROS accumulation increasing, the IC50 of these four drugs also increases (Figure 5B, *p* < 0.0001). Then, we hypothesized that ROS, as a second messenger, can alter the pathway activation and thus change the IC50 of specific targeting drugs. Considering that glioma was characterized by ROS accumulation and generation, we selected two well-established glioma cell lines (U87 and LN229) to conduct *in vitro* studies. THP-1, a peripheral monocyte cell line was also cultured to further validate our findings. By supplementing cells with exogenous H_2_O_2_, NF-κB and ERK were activated in these three cell lines; the STAT3 pathway was inhibited in glioma U87 and LN229 cell lines while activated in the THP-1 cell, whereas the AKT pathway was activated in the U87 and THP-1 cell lines but inhibited in LN229 cell line (Figure 5C). Thus, the drug targeting oncogenic pathway activity could be modulated by ROS.

On the other hand, eight out of the 11 drugs (methotrexate, vorinostat, I-BET-762, NPK76-II-21-1, Navitoclax, PHA-793887, AT-7519, and TPCA-1) with the highest correlation to index II were cell cycle or chromosome modification inhibitors. With oxidative stress degree deepening, normal cell cycle or DNA modification process would be affected and decrease the IC50 of the eight drugs (Figure 5B, *p* < 0.0001).

Next, we proposed a ROS combining strategy to decrease the IC50s of the above-mentioned drugs. We set the maximum safety concentrations of each drug as a threshold to determine whether combined approaches should be used. When a drug's IC50 correlated with index I and was greater than the safety concentration, ROS scavenging compounds, *e*. *g*., NAC, was used in combination to abolish ROS activating effects on oncogenic pathways (Figure 5D). If an index II correlated drug's IC50 was greater than the safety concentration, anti-oxidant inhibitors could be combined to abolish normal ROS responses and force cells into oxidative stress (Figure 5E). Therefore, regulating ROS metabolism in this manner may be a promising approach to improving drug efficacy and expanding drug applications.

### Drug Efficacy Can Be Modulated by ROS Administration

**Figure 6:**
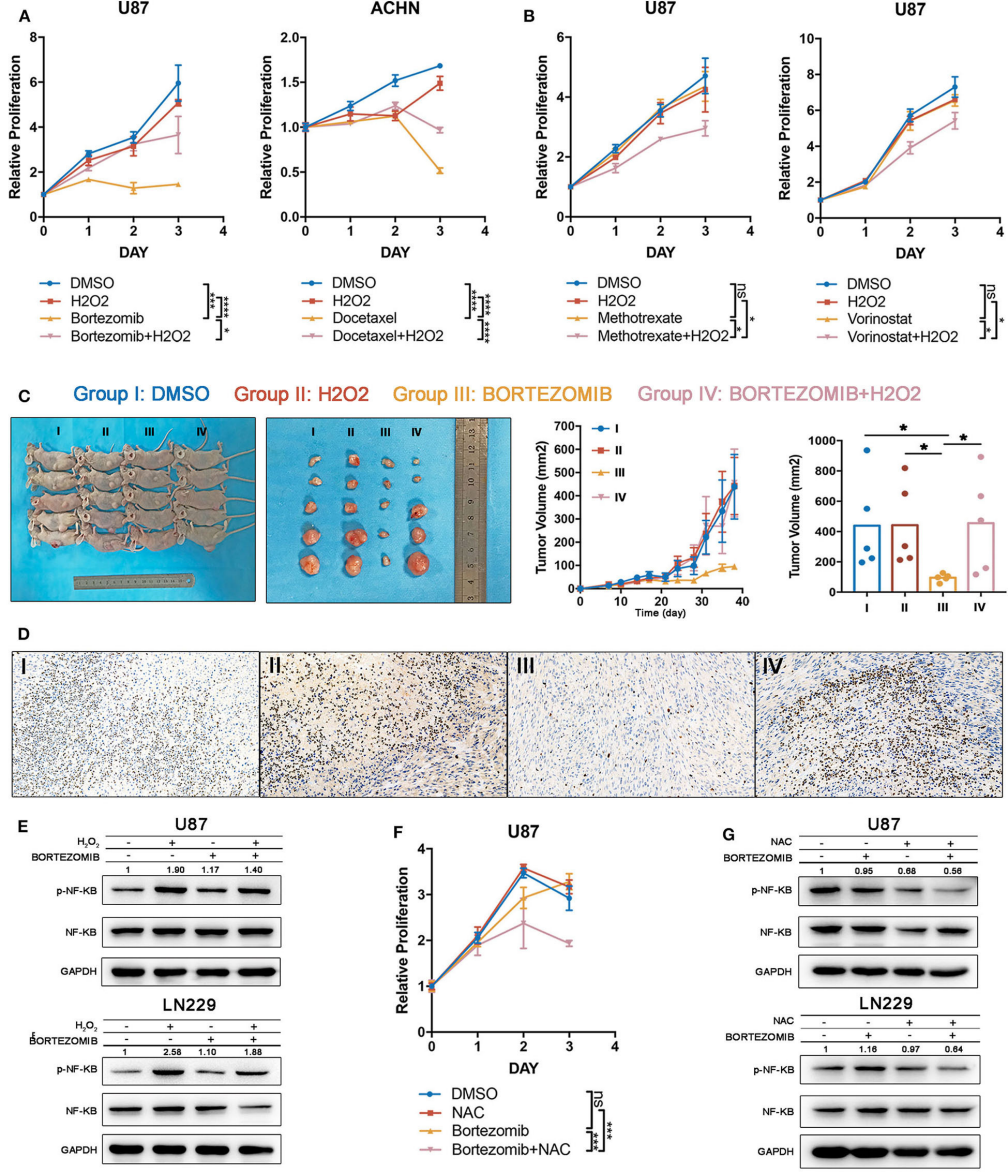
Drug efficacy can be modulated by targeting reactive oxygen species (ROS) metabolism. **(A)** The drug efficacies of bortezomib and docetaxel were assessed by adding a low concentration of H_2_O_2_ together as assessed by cell proliferation assay. The antitumor efficacy of these two drugs was diminished by exogenous ROS. Error bars indicate the mean ± SD; ns = no significance, *p < 0.05, **p < 0.01, ***p < 0.001, ****p < 0.0001. **(B)** The drug efficacies of methotrexate and vorinostat were increased by adding a high concentration of H_2_O_2_ together as assessed by cell proliferation assay. Error bar indicate the mean ± SD; *p < 0.05, **p < 0.01, ***p < 0.001, ****p < 0.0001. **(C)** The tumor inhibitory effects of bortezomib were abolished by exogenous low H_2_O_2_ concentrations in a U87 subcutaneous xenograft model; *p < 0.05. Error bars indicate the mean ± SEM. **(D)** Immuno-histochemical staining for Ki67 shows that the proliferative inhibition of bortezomib was rescued by exogenous low H_2_O_2_ concentrations. **(E)** The NF-κB inhibitory effects of bortezomib were rescued by H_2_O_2_ in U87 and LN229 cell lines. **(F)** Half-dosage of bortezomib could be sensitized by NAC in U87 cell lines; ***p < 0.001. **(G)** Half-dosage of bortezomib together with NAC could inhibit NF-κB pathway in U87 and LN229 cell lines.

To test the accuracy of our combining strategy, four drugs in phase III clinical trials or already with broad clinical uses were selected for further investigation. Bortezomib (NF-κB inhibitor) and docetaxel (PI3K/AKT inhibitor) demonstrated significant correlations with index I, indicating that ROS accumulation may abrogate the anti-tumor effects of these drugs. Methotrexate (DNA replication inhibitor) and vorinostat (HDAC inhibitor) generated significant correlations with index II, indicating that oxidative stress may augment the anti-tumor effects of these drugs (Supplementary Table 9). Cell lines were filtered out according to the drug IC50. Glioma cells were preferentially used for studies, attributing to the above-mentioned importance of ROS metabolism in glioma. Supposedly sensitive and resistant cell lines (defined whether the putative IC50 could exceed the maximum screening concentration provided by the GDSC database) were cultured to test drugs which were correlated with indexes I and II, respectively. With low H_2_O_2_ supplied together simultaneously, the supposedly sensitive cell lines became resistant to bortezomib and docetaxel (Figure 6A), whereas high H_2_O_2_ concentrations sensitized cell lines to methotrexate and vorinostat when being supplied simultaneously (Figure 6B). Besides that, to further investigate the anti-tumor effect of bortezomib in glioma cell lines related to ROS, we also conducted the antagonized experiment using two other glioma cell lines, U251 and LN229. Similar to the U87 cell line, low concentrations of H_2_O_2_ could diminish the anti-tumor effect in U251 and LN229 cell lines (Supplementary Figures 4A,C).

We next validated our findings in an *in vivo* xenograft experiment. Bortezomib is effective in reducing tumor burden by targeting the NF-κB pathway (32). However, the latest clinical trial showed that bortezomib failed to prolong the survival time of glioma patients (ClinicalTrials.gov: NCT00512798). Here we observed that glioma was characterized by high ROS accumulation; therefore, it was reasonable to test whether the treatment failure for bortezomib was derived from excessive ROS. Exogenous low H_2_O_2_ concentrations were used to mimic elevated ROS levels in glioma tissue. We found that although bortezomib inhibited tumor growth, the administration of H_2_O_2_ fully abolished the established antitumor effects (Figures 6C,D). Immuno-histochemical staining for Ki67 indicated that bortezomib suppressed tumor cell proliferation, which was rescued by supplementing with H_2_O_2_ (Figure 6D). Consistent with *in vivo* experiments, although bortezomib could inhibit NF-KB activation partially with exogenous H_2_O_2_ added, the activation level was still higher than no exogenous was added (Figure 6E).

Standard dosage of bortezomib treatment will increase the hazard ratio of serious adverse events (grades 3 and 4) (35), which urges us to explore how to achieve therapeutic efficacy with reduced dosage. Therefore, we tried to investigate whether half-dose bortezomib can inhibit tumor growth when combined with NAC, which decreases ROS accumulation. The cell viability assay has shown that although U87, U251, and LN229 were resistant to half-dose bortezomib, the resistance could be reverse by supplementing NAC (Figure 6F, Supplementary Figures 4B,D). We observed that half-dose bortezomib failed to inhibit the NF-KB pathway, while the combined administration of NAC and half-dose bortezomib effectively abolish NF-κB activation (Figure 6G). These results together suggest that ROS metabolism status plays an important role in modulating drug efficacy, which is meaningful in sensitizing drugs and reducing dosage.

### Potential Biomarkers Related to the ROS Indexes

Finally, to promote the clinical translation of our method, we tried to find the potential biomarkers related to our five ROS indexes. Two strategies were applied to achieve this goal. Firstly, protein–protein interaction analysis was used to define the hub genes of each index. Next, the correlation between hub gene expression and index value was calculated. A hub gene with |*R*| larger than 0.2 was considered to be a potential biomarker. All the biomarkers identified are summarized in Table 1.

**Table 1:** Potential Biomarkers Related to five ROS Indexes.

**Index**	**Positive direction biomarker**	**Negative direction biomarker**
Index I	PINK1	CYBA, SOD3, NOS3, NCF2, NOX4, TGFB1, EDN1, ICAM1, IL6
Index II	NA	PINK1, GPR37
Index III	TGFB1, EDN1, ICAM1, IL6, PTGS2, INS	BNIP3
Index IV	TLR4, EGFR	NA
Index V	PGD, XYLB, TALDO1, TKT	CYBA, NCF1, NCF2

## Discussion

Aberrant ROS production and scavenging are key tumor characteristics for maintaining optimal redox homeostasis (4). The effects of antitumor therapies may be abolished by powerful anti-oxidant capabilities within tumors, but they can be rescued by combinatorial strategies targeting redox homeostasis (12, 36). Therefore, the comprehensive evaluation of ROS status is important for understanding tumor biology and the development of effective therapies. However, current methods fail to reflect the interior redox balance because of methodological limitations and are therefore insufficient to fully guide ROS metabolic modulating strategies in clinical situations. In this study, we developed a new method to comprehensively evaluate intrinsic ROS biotransformation abilities, whereby we reclassified tumor patients and established therapeutic regimens according to ROS metabolic status.

The comprehensive evaluation of ROS metabolism is essential to investigate homeostatic redox balance. Current methods mainly focus on directly measuring ROS concentrations, relying on fluorescent ROS probes. By using these methods, specimens must be incubated with probes for a period of time, which may dramatically alter ROS levels from endogenous levels (19). In addition, probes are limited to detecting ROS in single cells, whereas tumors are characterized by high cellular heterogeneity and a complex microenvironment. The assessment of key enzymes in ROS metabolic processes, such as superoxide dismutase, GPX, and PRDX, is also widely used to characterize ROS (20). However, ROS comprises a group of oxygen derivatives, which are constantly generated and scavenged in subcellular compartments by fluctuating cascades; therefore, detecting specific enzymes does not reflect cascading ROS metabolism. To comprehensively understand ROS metabolic status in cancer, some studies have attempted to simultaneously detect these cascading ROS metabolism pathways (7, 27). However, harsh experimental conditions often limit their translation to clinical practice. In recent years, evidence has suggested that gene signatures are reliable in evaluating biological phenotypes (28, 37). Here we summarized ROS metabolic gene signatures and developed five ROS indexes. These indexes reflected ROS metabolism, including (1) ROS accumulation, (2) oxidative stress levels, (3, 4) biosynthetic and scavenging abilities, and (5) subcellular origins. Our approach has several advantages: firstly, both cellular status and intrinsic ability are evaluated; secondly, complete ROS metabolism is evaluated, rather than single reactions, and thirdly, our approach has huge practical potential when aligned with sequencing technologies and other proteomic, genomic, and metabolomic developments.

Metabolic alterations are important for tumorigenesis and tumor development (38). However, we have little knowledge on ROS metabolism in determining human cancer outcomes. Here we observed that ROS indexes exerted different prognostic values, depending on the cancer type. These findings are consistent with previous studies showing that important ROS enzymes act as bidirectional prognostic indicators in different cancers (39, 40). ROS accumulation, represented by index I, was positively and negatively correlated with survival times in LGG and KIRC, respectively. This observation may derive from their distinct basal ROS metabolism status (Figure 2A). LGG was characterized by the highest levels of ROS accumulation; therefore, further ROS production and enrichment may alter the subtle redox homeostasis, thus inducing programmed cell death. In contrast, KIRC had the lowest ROS accumulation; thus, an enriched ROS status could facilitate aggressive progression. These findings highlighted the importance of intrinsic ROS status to help guide and modulate ROS metabolism.

Increasing evidence has suggested that different cancer types share substantial biological similarities (41, 42). Reclassifying tumors, regardless of different cancer types, may help redesign more effective clinical trials (43, 44). Here we classified tumor samples into eight clusters based on ROS metabolic indexes. Distinct ROS metabolic phenotypes were observed among our clusters. Notably, cluster I was characterized by the highest levels of ROS accumulation and generation. This cluster was primarily composed of glioma (79.3%) tumors, and most glioma samples were classified to cluster I (95.8%). However, the composition of other clusters was heterogeneous, indicating that tumors originating from other organs tended to share substantial ROS metabolic similarities. Notably, GBM and LGG had equal ROS metabolism levels, distinguishing them from other tumor samples. The brain is physically characterized by ROS enrichment, as it consumes up to 20% of the body's oxygen. High tumor cell proportions and characteristic hypoxic microenvironments further facilitate glioma ROS production (45, 46). Multi-omics analyses suggested that recurrent oncogenic molecular events in cluster I, *e*. *g*., IDH1 mutation (47) and EGFR amplification (48), reprogrammed tumors to a ROS promoting status. Therefore, ROS modulating strategies against gliomas are reasonably trans-applied to patients with abundant ROS accumulation or harboring multi-omics features to glioma. For cancers like ESCA and KIRC, patients with high ROS accumulation have also shown reduced survival time compared to their counterparts (Figure 3G).

During tumor therapy, additional ROS may be generated to disrupt an already established tumor redox homeostasis (10). Here we established a ROS-based drug efficacy predicting approach. ROS levels and cellular responses were highlighted due to their substantial roles in regulating antitumor drug responses. Of these drugs, pathway inhibitors were antagonized by low supplemented ROS levels, while chromosome or cell cycle targeting drugs were augmented by damaging normal cellular responses, which was consistent with previous reports (4, 13, 49) and verified by our studies. NF-κB signaling is accepted as an oncogenic pathway; however, targeting NF-κB by bortezomib failed to prolong patient survival times in a recent clinical trial, including patients with glioma, melanoma, and lymphoma (32). We found that the antitumor effects and NF-κB inhibition of bortezomib was abrogated by increasing ROS levels. NF-κB pathway can inhibit Nrf2 pathway and increase the ROS level in the tumor microenvironment, which might provide an explanation in bortezomib resistance. In our study, we found one possible measure to sensitize bortezomib by adding NAC. These findings provide novel clues about targeting treatment resistance. ROS establishes a complex regulatory network by acting as a second messenger (7). Even when inhibitors specifically block some key nodes, ROS rescues or bypasses the inhibition to support tumor progression (50). Therefore, modulating ROS metabolism will be helpful in improving targeted treatment outcomes. Our ROS indexes may equally provide references for evaluating ROS status, selecting suitable patients, and predicting treatment responses.

Taken together, we developed a robust method to provide an overall assessment of ROS metabolism. Using this method, tumor samples were reclassified in terms of ROS status, which provided novel clinical and molecular insights. We established a ROS-based drug efficacy evaluation network, suggesting that ROS modulation is helpful in improving treatment responses and expanding drug applications. Taken together, this study highlights the importance of ROS homeostasis in human cancers and provides novel information on developing diagnostic models and treatment strategies.

## Data Availability Statement

All datasets generated for this study are included in the article/Supplementary Material.

## Ethics Statement

The animal study was reviewed and approved by The Ethics Committee of The First Hospital of China Medical University.

## Author Contributions

SS, ZY, JW, WC, and AW contributed to the study conceptualization. ZY, XL, and CC contributed to data curation. AW, WC, PC, and CC contributed to funding acquisition. JW, CZo, QG, and CZhs took charge of the experiments. GG, LC, and PC took charge of the methodology. TL, CZo, and QG contributed to project administration. WC and AW supervised the study and took charge of the review and editing. SS took charge of validation. SS, ZY, and TL contributed to writing the original draft. All authors contributed to the article and approved the submitted version.

## Conflict of Interest

The authors declare that the research was conducted in the absence of any commercial or financial relationships that could be construed as a potential conflict of interest.
