# Supplementary material for: Characterization of ROS Metabolic Equilibrium Reclassifies Pan-Cancer Samples and Guides Pathway Targeting Therapy
Source: Front Oncol. 2020 Oct 20;10:581197. doi: 10.3389/fonc.2020.581197 (PMC7606976; doi:10.3389/fonc.2020.581197)
Supplement: Supplementary file 1 [file Data_Sheet_1.PDF]

Table S1 17 Well-established Gene Sets from Msigdb Database involving in ROS metabolism

GO ID	GO Name	Ref Website
GO:1903409	GO_REACTIVE_OXYGEN_SPECIES_BIOSYNTHETIC_PROCESS	http://www.broadinstitute.org/gsea/msigdb/cards/GO_REACTIVE_OXYGEN_SPECIES_BIOSYNTHETIC_PROCESS
GO:0072593	GO_REACTIVE_OXYGEN_SPECIES_METABOLIC_PROCESS	http://www.broadinstitute.org/gsea/msigdb/cards/GO_REACTIVE_OXYGEN_SPECIES_METABOLIC_PROCESS
GO:1903428	GO_POSITIVE_REGULATION_OF_REACTIVE_OXYGEN_SPECIES_BIOSYNTHETIC_PROCESS	http://www.broadinstitute.org/gsea/msigdb/cards/GO_POSITIVE_REGULATION_OF_REACTIVE_OXYGEN_SPECIES_BIOSYNTHETIC_PROCESS
GO:1903427	GO_NEGATIVE_REGULATION_OF_REACTIVE_OXYGEN_SPECIES_BIOSYNTHETIC_PROCESS	http://www.broadinstitute.org/gsea/msigdb/cards/GO_NEGATIVE_REGULATION_OF_REACTIVE_OXYGEN_SPECIES_BIOSYNTHETIC_PROCESS
GO:2000378	GO_NEGATIVE_REGULATION_OF_REACTIVE_OXYGEN_SPECIES_METABOLIC_PROCESS	http://www.broadinstitute.org/gsea/msigdb/cards/GO_NEGATIVE_REGULATION_OF_REACTIVE_OXYGEN_SPECIES_METABOLIC_PROCESS
GO:2000379	GO_POSITIVE_REGULATION_OF_REACTIVE_OXYGEN_SPECIES_METABOLIC_PROCESS	http://www.broadinstitute.org/gsea/msigdb/cards/GO_POSITIVE_REGULATION_OF_REACTIVE_OXYGEN_SPECIES_METABOLIC_PROCESS
GO:0006123	GO_MITOCHONDRIAL_ELECTRON_TRANSPORT_CYTOCHROME_C_TO_OXYGEN	http://www.broadinstitute.org/gsea/msigdb/cards/GO_MITOCHONDRIAL_ELECTRON_TRANSPORT_CYTOCHROME_C_TO_OXYGEN
GO:0016175	GO_SUPEROXIDE_GENERATING_NADPH_OXIDASE_ACTIVITY	http://www.broadinstitute.org/gsea/msigdb/cards/GO_SUPEROXIDE_GENERATING_NADPH_OXIDASE_ACTIVITY
GO:0042743	GO_HYDROGEN_PEROXIDE_METABOLIC_PROCESS	http://www.broadinstitute.org/gsea/msigdb/cards/GO_HYDROGEN_PEROXIDE_METABOLIC_PROCESS
GO:0042744	GO_HYDROGEN_PEROXIDE_CATABOLIC_PROCESS	http://www.broadinstitute.org/gsea/msigdb/cards/GO_HYDROGEN_PEROXIDE_CATABOLIC_PROCESS
GO:0019321	GO_PENTOSE_METABOLIC_PROCESS	http://www.broadinstitute.org/gsea/msigdb/cards/GO_PENTOSE_METABOLIC_PROCESS
GO:0000302	GO_RESPONSE_TO_REACTIVE_OXYGEN_SPECIES	http://www.broadinstitute.org/gsea/msigdb/cards/GO_RESPONSE_TO_REACTIVE_OXYGEN_SPECIES
GO:1901031	GO_REGULATION_OF_RESPONSE_TO_REACTIVE_OXYGEN_SPECIES	http://www.broadinstitute.org/gsea/msigdb/cards/GO_REGULATION_OF_RESPONSE_TO_REACTIVE_OXYGEN_SPECIES
GO:0034614	GO_CELLULAR_RESPONSE_TO_REACTIVE_OXYGEN_SPECIES	http://www.broadinstitute.org/gsea/msigdb/cards/GO_CELLULAR_RESPONSE_TO_REACTIVE_OXYGEN_SPECIES
GO:1903426	GO_REGULATION_OF_REACTIVE_OXYGEN_SPECIES_BIOSYNTHETIC_PROCESS	http://www.broadinstitute.org/gsea/msigdb/cards/GO_REGULATION_OF_REACTIVE_OXYGEN_SPECIES_BIOSYNTHETIC_PROCESS
GO:2000377	GO_REGULATION_OF_REACTIVE_OXYGEN_SPECIES_METABOLIC_PROCESS	http://www.broadinstitute.org/gsea/msigdb/cards/GO_REGULATION_OF_REACTIVE_OXYGEN_SPECIES_METABOLIC_PROCESS
GO:1901032	GO_NEGATIVE_REGULATION_OF_RESPONSE_TO_REACTIVE_OXYGEN_SPECIES	http://www.broadinstitute.org/gsea/msigdb/cards/GO_NEGATIVE_REGULATION_OF_RESPONSE_TO_REACTIVE_OXYGEN_SPECIES
