# Supplementary material for: Characterization of ROS Metabolic Equilibrium Reclassifies Pan-Cancer Samples and Guides Pathway Targeting Therapy
Source: Front Oncol. 2020 Oct 20;10:581197. doi: 10.3389/fonc.2020.581197 (PMC7606976; doi:10.3389/fonc.2020.581197)
Supplement: Supplementary file 3 [file Data_Sheet_3.PDF]

Table S3 Detailed calculating formula of ROS Indexes

	Representation	Calculation Formula
Index I	Accumulation of ROS	Index I= (GO 1 score *GO 3 score * GO 5 score)$^{1/3}$ / (GO 2 score * GO 4 score * GO6 score)$^{1/3}$
Index II	Oxidative Stress	Index II=GO 7 score / GO 8 score
Index III	Scavenging Ability of ROS	Index III=GO 6 score / GO 5 score
Index IV	Biosynthetic Ability of ROS	Index IV=GO 3 score / GO 4 score
Index V	Subcellular Origin of ROS	Index V= GO 9 score / GO 10 score

GO 1=GO_REACTIVE_OXYGEN_SPECIES_BIOSYNTHETIC_PROCESS
GO 2=GO_REACTIVE_OXYGEN_SPECIES_METABOLIC_PROCESS
GO 3=GO_POSITIVE_REGULATION_OF_REACTIVE_OXYGEN_SPECIES_BIOSYNTHETIC_PROCESS
GO 4=GO_NEGATIVE_REGULATION_OF_REACTIVE_OXYGEN_SPECIES_BIOSYNTHETIC_PROCESS
GO 5=GO_NEGATIVE_REGULATION_OF_REACTIVE_OXYGEN_SPECIES_METABOLIC_PROCESS
GO 6=GO_POSITIVE_REGULATION_OF_REACTIVE_OXYGEN_SPECIES_METABOLIC_PROCESS
GO 7=GO_REGULATION_OF_RESPONSE_TO_REACTIVE_OXYGEN_SPECIES
GO 8=GO_NEGATIVE_REGULATION_OF_RESPONSE_TO_REACTIVE_OXYGEN_SPECIES
GO 9=GO_PENTOSE_METABOLIC_PROCESS
GO 10=GO_SUPEROXIDE_GENERATING_NADPH_OXIDASE_ACTIVITY

Kowald et.al (1)measured several coefficients in the ROS cascade metabolic process in biology. According to their research, biological process overwhelms other process in ROS bio-metabolism. Therefore, only biological process was considered in our indexes. After simplifying their formula to $\frac{\mathrm{dROS}}{\mathrm{dt}} = \mathrm{k1} * (\mathrm{ROS\ generating\ activity})\text{-}\mathrm{k2} * (\mathrm{antioxidant\ enzyme\ activity}) * \mathrm{ROS}$, ROS accumulation (Index I) is can be acquired as an integral of metabolic ability*primitive ROS content in a period of time. Metabolic ability can be seen as the ratio between ROS biosynthetic ability (Index IV) and ROS scavenging ability (Index III). Primitive ROS content can be seen as the ratio between ROS biosynthetic process and ROS metabolic process. Thereby, ROS accumulation=$\sqrt[3]{\frac{\mathrm{Index\ IV}}{\mathrm{Index\ III}} * \frac{\mathrm{ROS\ biosynthetic\ process}}{\mathrm{ROS\ metabolic\ process}}}$. The ratio between positive and negative regulation of

ROS metabolism was used to reflect scavenging ability of ROS (Index III). The ratio between positive and negative regulation of ROS biosynthesis was used to reflect biosynthetic ability of ROS (Index IV). Besides, we think the degree of oxidative stress that ROS makes (Index II) and the subcellular origin of ROS (Index V) are also important. The ratio between ROS response process and negative response process was used to reflect oxidative stress degree (Index II). There are mainly two ROS origin in cellular, the MIT pattern or cytoplasmic pattern. During cytoplasmic pattern, NADPH is consumed by NOXs to generate ROS(①)(2). NADPH also plays an important role in ROS scavenging process by supplementing reducing substances （②）. The total NADPH consumed in ROS metabolism is equal to ①+②. It is clearly that a high (①+②)/② value means that less NADPH is consumed by NOXs which means more ROS is generated from MIT. Pentose metabolic process is the major source of NADPH in cells which can be used to resemble total NADPH. Therefore, the ratio between pentose metabolism and NADPH consuming oxidases activity is used to evaluate the subcellular origin of ROS (Index V).

Geometric mean is used to score each gene sets.

1. Kowald A, Hamann A, Zintel S, Ullrich S, Klipp E, Osiewacz HD. A systems biological analysis links ROS metabolism to mitochondrial protein quality control. Mech Ageing Dev. 2012;133(5):331-7.
2. Hegedus C, Kovacs K, Polgar Z, Regdon Z, Szabo E, Robaszkiewicz A, et al. Redox control of cancer cell destruction. Redox Biol. 2018;16:59-74.
