# Supplementary figures and images for: Characterization of ROS Metabolic Equilibrium Reclassifies Pan-Cancer Samples and Guides Pathway Targeting Therapy
Source: Front Oncol. 2020 Oct 20;10:581197. doi: 10.3389/fonc.2020.581197 (PMC7606976; doi:10.3389/fonc.2020.581197)

# Figure S1

## A

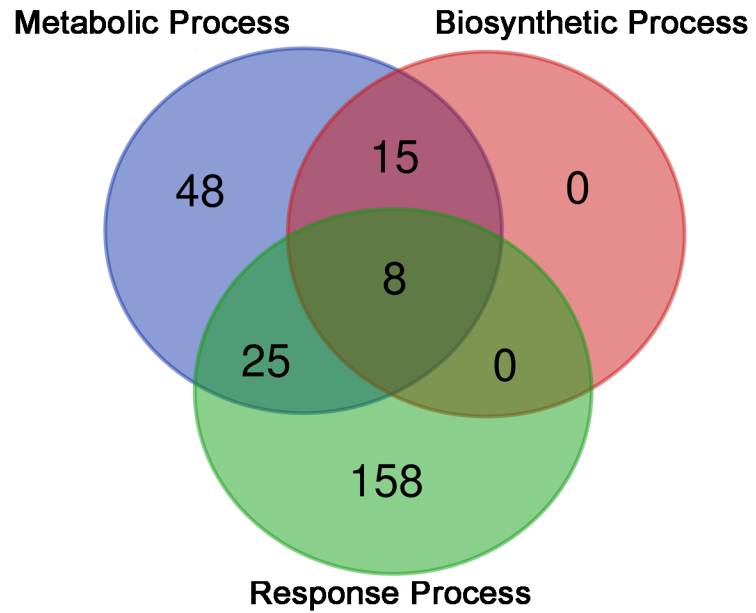

## B

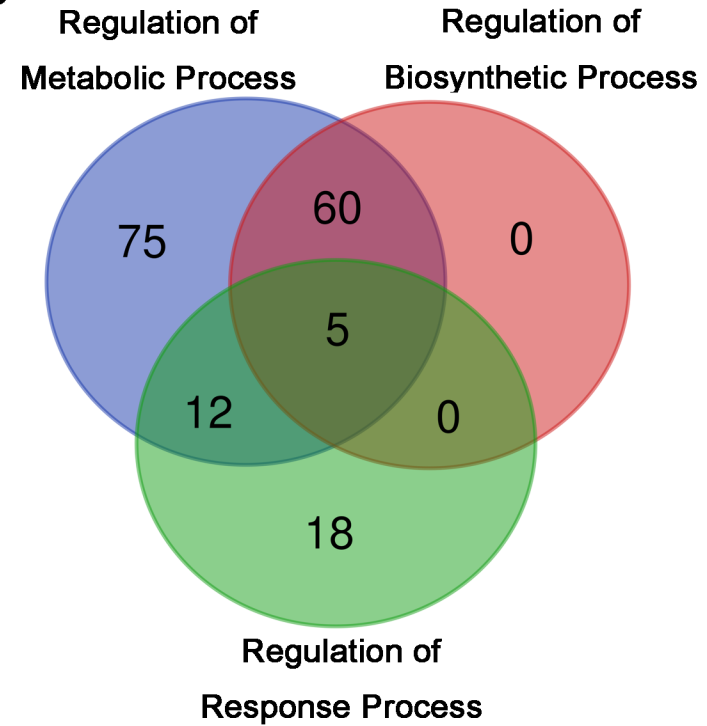

## C

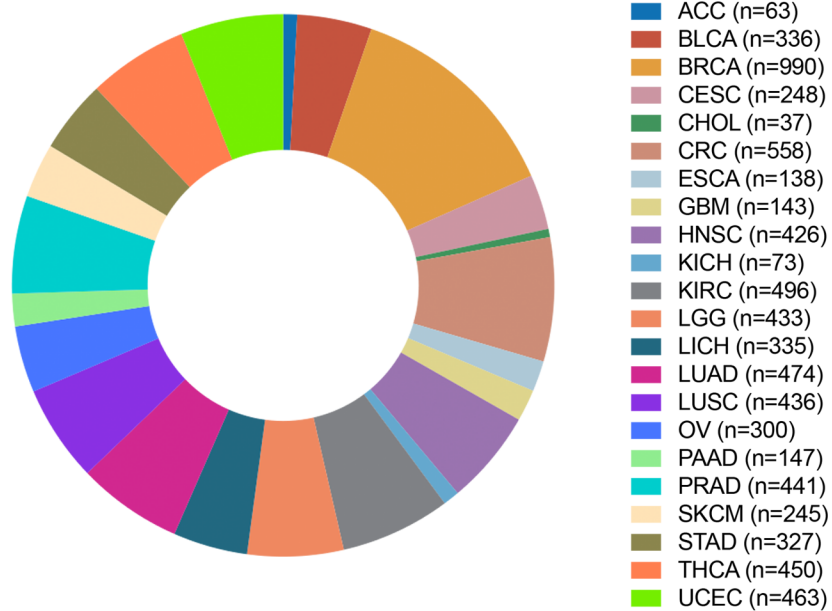

Supplement: Supplementary file 10 [file Image_1.PDF]

# Figure S2

## A

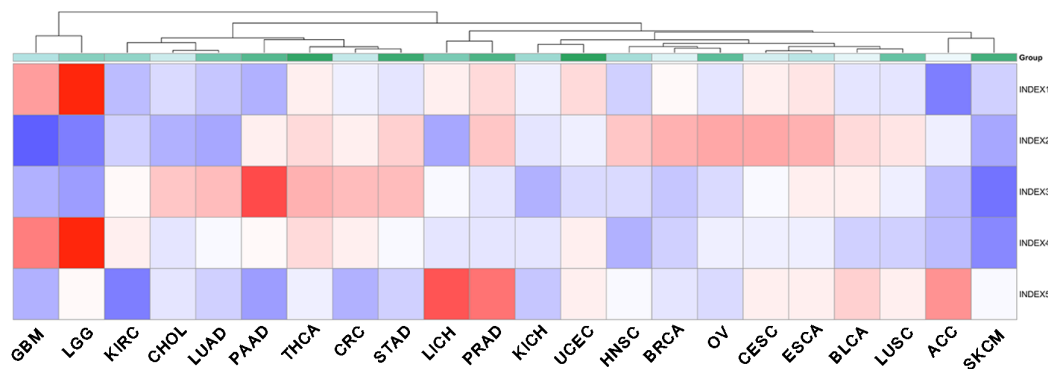

## B

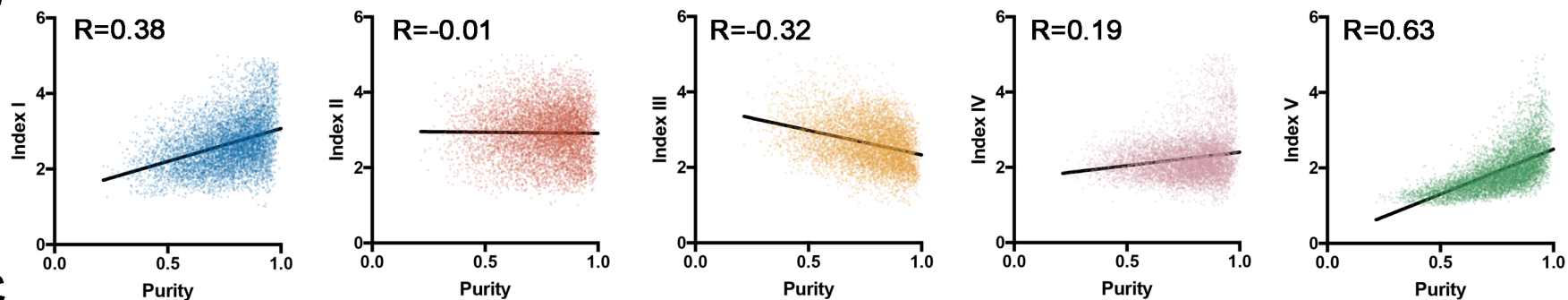

## C

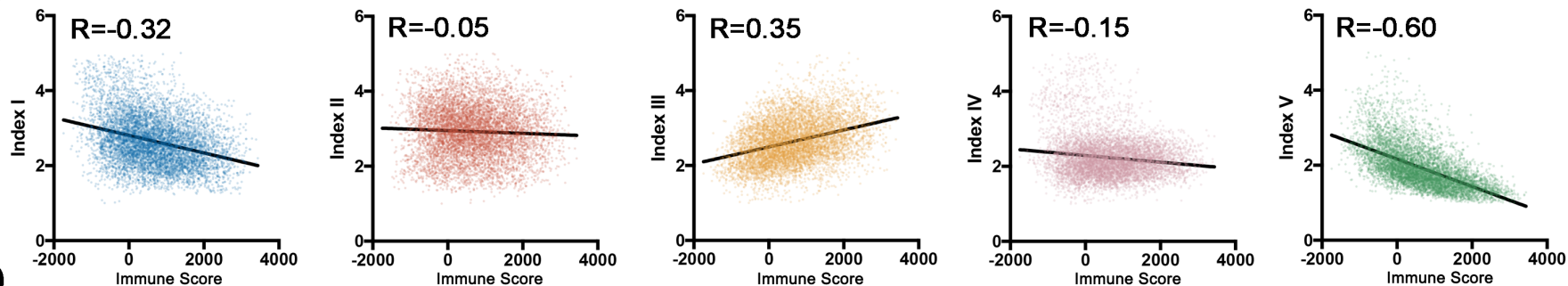

## D

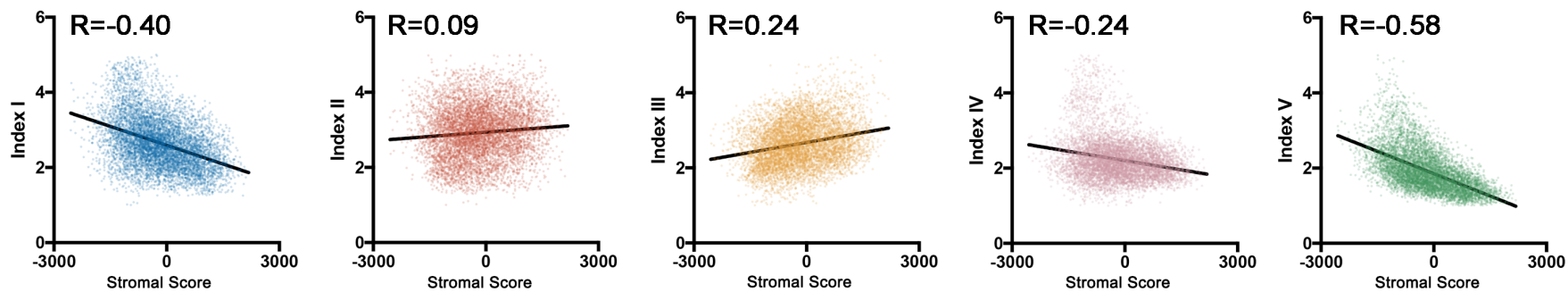

Supplement: Supplementary file 11 [file Image_2.PDF]

Figure S3

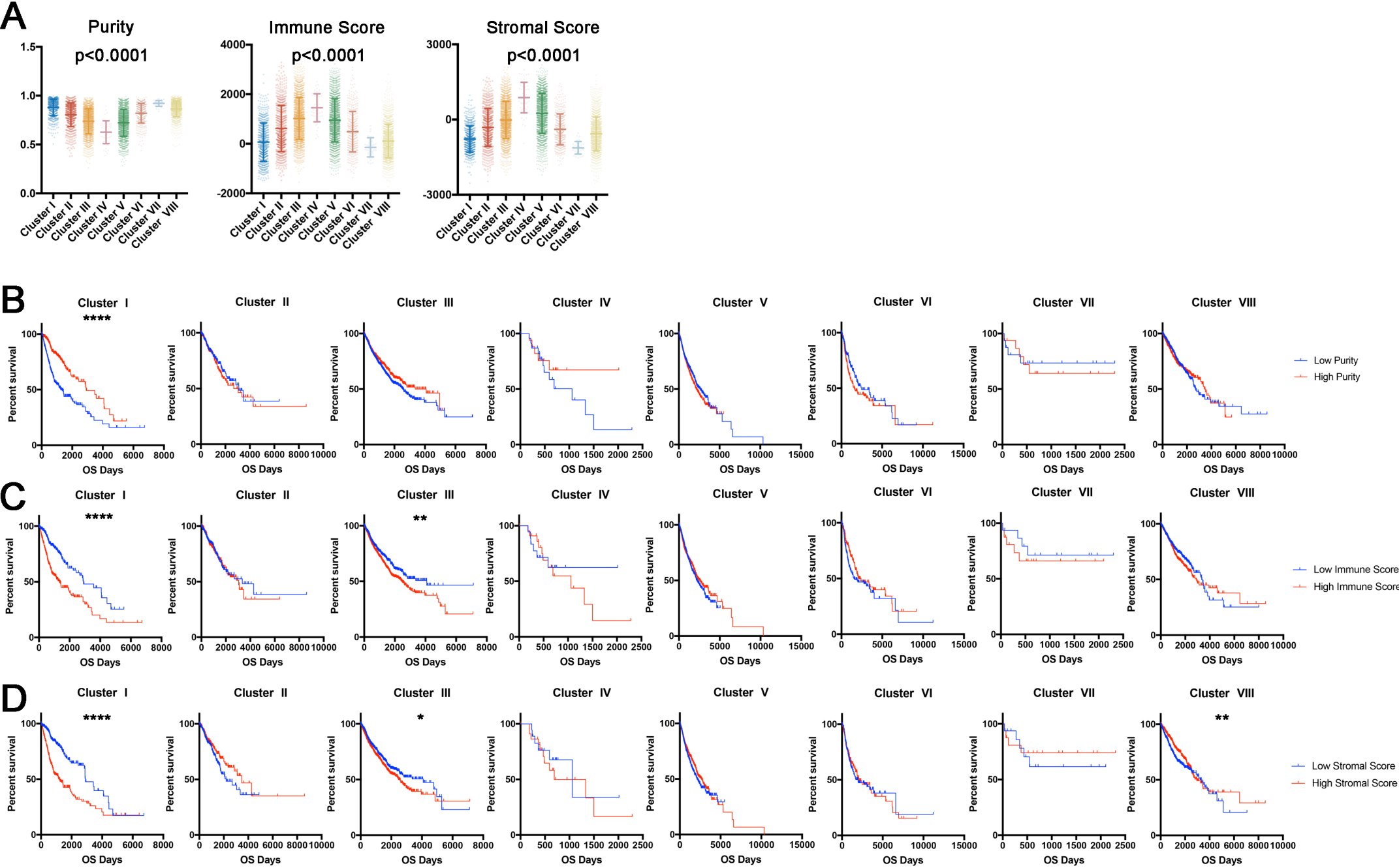

Supplement: Supplementary file 12 [file Image_3.PDF]

# Figure S4

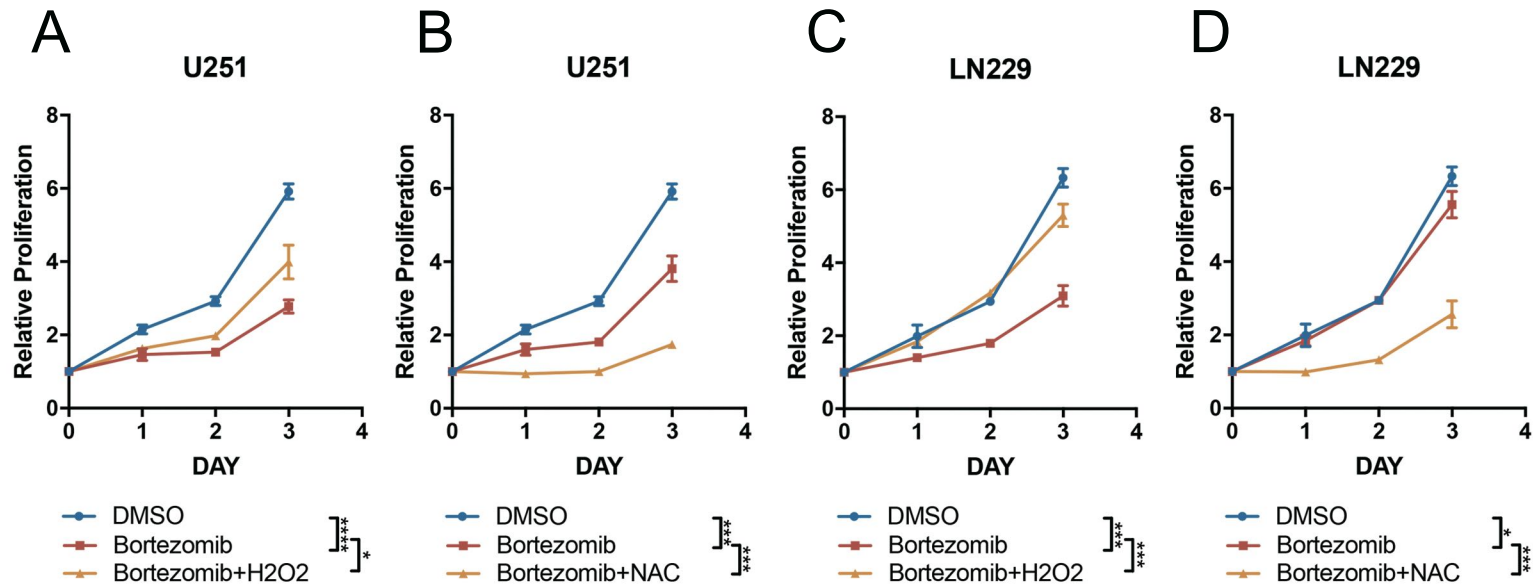

Supplement: Supplementary file 13 [file Image_4.PDF]
