# Supplementary material for: Characterization of ROS Metabolic Equilibrium Reclassifies Pan-Cancer Samples and Guides Pathway Targeting Therapy
Source: Front Oncol. 2020 Oct 20;10:581197. doi: 10.3389/fonc.2020.581197 (PMC7606976; doi:10.3389/fonc.2020.581197)
Supplement: Supplementary file 14 [file Presentation_1.PDF]

Supplementary Material

1 Supplementary Figures and Tables

1.1 Supplementary Figures

Supplementary Figure 1. Additional information related to ROS metabolism across different cancer types.

A. Venn diagram showing intersections between ROS metabolic processes, ROS biosynthetic processes and ROS response processes.

B. Venn diagram showing intersections between regulation of ROS metabolic processes, regulation of ROS biosynthetic processes and regulation of ROS response processes.

C. Sample composition in this study after filtering (cancer types = 22, n = 7559).

Supplementary Figure 2. The relationship between ROS indexes and microenvironment parameters.

A. Clustering results of 22 cancer types based on ROS metabolism related Indexes.

B. Associations between index values and purity across eight ROS clusters as calculated by Pearson correlation.

C. Associations between index values and leukocyte scores across eight ROS clusters as calculated by Pearson correlation.

D. Associations between index values and stromal scores across eight ROS clusters as calculated by Pearson correlation.

Supplementary Figure 3. Tumor microenvironment analysis across eight ROS clusters.

A. The distribution of tumor purity, immune scores and stromal scores among eight ROS clusters.
B. Survival differences between high purity groups and low purity groups among eight ROS clusters, * $p < 0.05$, **** $p < 0.0001$.
C. Survival differences between high leukocyte score groups and low leukocyte score groups among eight ROS clusters, ** $p < 0.01$, **** $p < 0.0001$.
D. Survival differences between high stromal score groups and low stromal score groups among eight ROS clusters, ** $p < 0.01$.

Supplementary Figure 4. Anti-tumor effect of Bortezomib could be modulated by ROS metabolism in U251 and LN229 cell lines.

A. The antitumor efficacy of Bortezomib was diminished by exogenous ROS (5 uM) added in U251 cell line, * $p < 0.05$, **** $p < 0.0001$.
B. Half-dosage of Bortezomib could be sensitized by NAC in U251 cell line, *** $p<0.001$.
C. The antitumor efficacy of Bortezomib was diminished by exogenous ROS added in LN229 cell line, *** $p < 0.001$.
D. Half-dosage of Bortezomib could be sensitized by NAC in LN229 cell line, * $p<0.05$, *** $p<0.001$.

1.2 Supplementary Tables

Supplementary Table 1. Seventeen established gene sets involving ROS metabolism (taken from the Msigdb database)

Supplementary Table 2. Prognostic value of ROS metabolism related gene sets across different cancer types

Supplementary Table 3. Calculation formulae for ROS Indexes

Supplementary Table 4. Index value of ROS Indexes in Cell lines

Supplementary Table 5. Correlation Indexes between five ROS Indexes

Supplementary Table 6. Univariate Cox analyses of ROS Indexes for different cancer types

Supplementary Table 7. Reclassification of samples after filtering outliers

Supplementary Table 8. Multi-omics alterations in ROS clusters

Supplementary Table 9. Correlations between AUC of GDSC drugs and ROS Indexes
